# Polysaccharide from *Salviae miltiorrhizae* Radix et Rhizoma Attenuates the Progress of Obesity-Induced Non-Alcoholic Fatty Liver Disease through Modulating Intestinal Microbiota-Related Gut–Liver Axis

**DOI:** 10.3390/ijms231810620

**Published:** 2022-09-13

**Authors:** Lixia Li, Xinting Lan, Xi Peng, Shuai Shi, Yanlin Zhao, Wentao Liu, Qihui Luo, Lanlan Jia, Bin Feng, Zhengli Chen, Yuanfeng Zou, Chao Huang

**Affiliations:** 1 Key Laboratory of Animal Disease and Human Health of Sichuan Province, College of Veterinary Medicine, Sichuan Agricultural University, Chengdu 611130, China; 2 Natural Medicine Research Center, College of Veterinary Medicine, Sichuan Agricultural University, Chengdu 611130, China; 3 Laboratory of Experimental Animal Disease Model, College of Veterinary Medicine, Sichuan Agricultural University, Chengdu 611130, China; 4 Animal Nutrition Institute, Sichuan Agricultural University, Chengdu 611130, China

**Keywords:** *Salviae miltiorrhizae* Radix et Rhizoma, polysaccharide, obesity, NAFLD, gut–liver axis, gut microbiota

## Abstract

Non-alcoholic fatty liver disease (NAFLD) is the most prevalent chronic liver disease worldwide, thus treatments for it have attracted lots of interest. In this study, the *Salviae miltiorrhizae* Radix et Rhizoma (SMRR) polysaccharide was isolated by hot water extraction and ethanol precipitation, and then purified by DEAE anion exchange chromatography and gel filtration. With a high-fat-diet-induced obesity/NAFLD mouse model, we found that consumption of the SMRR polysaccharide could remarkably reverse obesity and its related progress of NAFLD, including attenuated hepatocellular steatosis, hepatic fibrosis and inflammation. In addition, we also reveal the potential mechanism behind these is that the SMRR polysaccharide could regulate the gut–liver axis by modulating the homeostasis of gut microbiota and thereby improving intestinal function.

## 1. Introduction

Increasing evidence reveals a close interplay between the gut and liver named the “gut–liver axis”, which is implicated in both healthy physiology and disease conditions of the body. The “gut–liver axis” theory is based on the evidence that, on the one hand, bile acids, metabolites and immunoglobulins secreted by the liver enter the intestine through the bile ducts and participate in regulating intestinal function; on the other hand, the products of hosts and microorganisms metabolize endogenous and exogenous substrates which can translocate into the liver through the portal vein and affect liver function [1,2]. The homeostasis of the gut–liver axis is required for a healthy body, and an impaired gut barrier exposes the liver to gut-derived toxins, while dysregulated liver physiological processes implicated in gut dysfunction, which all contribute to disease, for example, ulcerative colitis (UC) and Crohn’s disease (CD) all corelate with liver dysfunctions [3,4,5]. Clinical evidence shows that in patients with inflammatory bowel disease (IBD), the prevalence of fatty liver disease is increased in most countries [6]. While the etiology and pathogenesis of IBD remain unclear, intestinal microbiota is thought to be one of the most important environmental factors that contribute to the development and progress of IBD [7]. In addition, crosstalk and interference between hepatic injures and intestinal lesions have been found in Crohn’s disease, liver cirrhosis and hepatic carcinoma [2,8,9,10]. Therefore, the intestine is emerging as an important target for liver disease treatment through the gut–liver axis, and signals implicated in gut immune response, epithelial permeability and metabolic processes are attracting growing interest.

As the most common chronic liver disease worldwide, non-alcoholic fatty liver disease (NAFLD) affects approximately 25% of the global adult population [11,12]. NAFLD is characterized by hepatic steatosis, progress of which will further result in inflammation and hepatocyte death-defined non-alcoholic steatohepatitis (NASH), NASH-related fibrosis/cirrhosis, hepatocellular carcinoma and liver failure [13,14]. Accumulating evidence reveals a close correlation between NAFLD and intestinal function. Increased prevalence of NAFLD was found in patients with inflammatory bowel disease [15], while defects in intestinal epithelial permeability promoted mice to develop severe histologic and pathologic features of NASH under obese condition [16]. Among all potential factors, gut microbiota is believed to be a key regulator affecting liver function. Defects in gut permeability or alterations in the composition of the microbiome could result in a translocation of gut microbes into the portal circulation and then to the liver [17,18]. Microbiota and its products can further drive proinflammatory gene expression and thus promote chronic hepatic inflammation, which is a major cause of NAFLD development and progress [19,20]. Therefore, screening and characterizing potential therapeutic molecules that balance gut microbiota to treat NAFLD is of great importance.

*Salviae miltiorrhizae* Radix et Rhizoma (SMRR), the roots and rhizomes of *Salvia miltiorrhizae* Bunge, is a well-known traditional Chinese medicine (TCM) named Danshen [21] and is widely used in Asian countries for the treatment of cardiovascular disease, hepatic injury, etc. [22]. In the past decades, the pharmacological effects of SMRR have been studied, isolating multiple bioactive compounds such as phenolic acids and tanshinone plus related quinone derivatives and protocatechualdehyde [21,23]. Recently, polysaccharides are emerging as critical components of SMRR with effective bioactivities in protecting against liver injures [24,25,26,27], but the underlying mechanisms are not well defined. In this study, we aim to evaluate whether polysaccharides from SMRR are of benefit for NAFLD, and to study the potential function of an SMRR polysaccharide in the gut–liver axis and its correlations with gut microbiota, providing evidence for further development and utilization of SMRR.

## 2. Results

### 2.1. Isolation, Purification and Characterization of SMRR Polysaccharide

**Figure 1:**
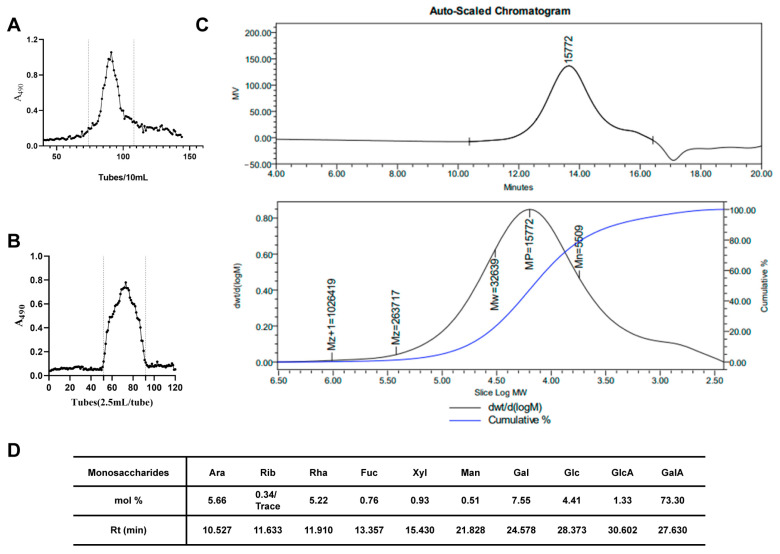
Isolation and characterization of SMRR polysaccharide. (**A**) The elution profile of crude SMRR polysaccharide on DEAE agarose gel FF (NaCl as eluent). A single fraction was obtained. (**B**) The elution profile of SMRR purification on Sepharose 6FF and SMRR was obtained. (**C**) The molecular weight determination of SMRR polysaccharide by gel permeation chromatography. (**D**) The monosaccharide composition of SMRR polysaccharide (mol %).

The crude polysaccharides from the roots and rhizomes of *Salviae miltiorrhizae* Radix et Rhizoma were obtained from water extracts after ethanol precipitation. The yield of crude SMRR polysaccharide was 21.5% (21.5 g crude SMRR polysaccharide from 100.0 g dried roots). The acidic polysaccharide fraction was obtained after ion exchange separation (Figure 1A) and gel filtration (Figure 1B), with a yield of 42.4% (42.4 mg from 100 mg crude SMRR polysaccharide).

The weight-average molecular weight (*M*_w_) of SMRR was 32.6 kDa, as determined by gel permeation chromatography, and a single symmetric peak was observed, as shown in Figure 1C. After methanolysis and GC analysis, the SMRR polysaccharide was shown to be mainly composed of galacturonic acid (GalA), arabinose (Ara), galactose (Gal), rhamnose (Rha) and glucose (Glc), with molar ratios of 17.9:1.3:1.7:1.2:1 (Figure 1D). Trace amounts of glucuronic acid (GlcA), xylose (Xyl), mannose (Man) and fucose (Fuc) were also found to be present in the SMRR polysaccharide, indicating that the SMRR polysaccharide was a typical pectic polysaccharide.

### 2.2. SMRR Polysaccharide Attenuates High-Fat-Diet-Induced Obesity

**Figure 2:**
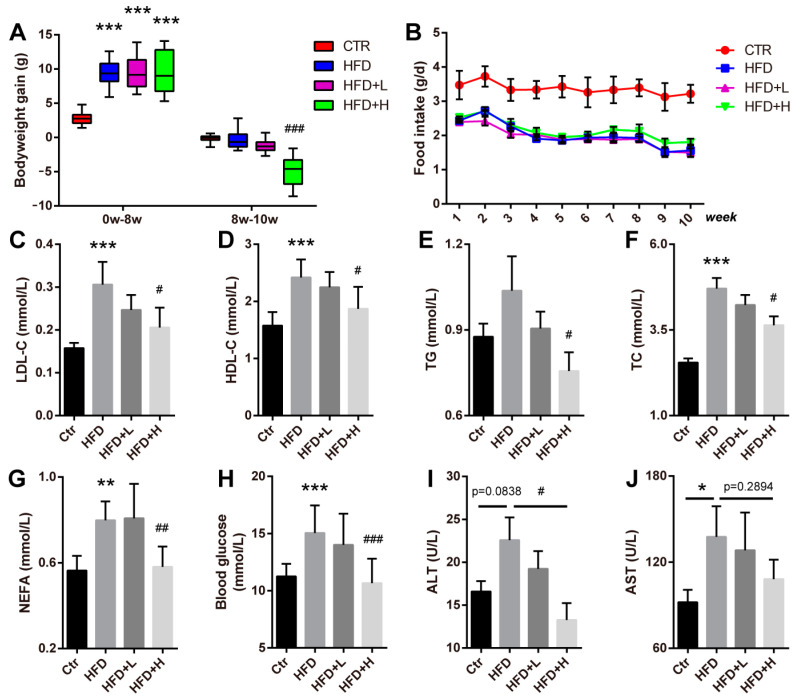
Anti-obesity effects of SMRR polysaccharide. (**A**) Quantification shows the bodyweight gain of the mice during the 0−8 w and 8−10 w in the experiment. Error bars indicate Mix to Max; *n* = 10 for CTR group, *n* = 12 for HFD group, *n* = 14 for HFD+L and HFD+H group; “*” stands for CTR vs. HFD, “#” stands for HFD vs. HFD+H; *** or ^###^ *p* < 0.001 by one-way ANOVA. (**B**) Quantification shows the food uptake of mice from different groups. Error bars indicate SD. (**C**–**H**) Quantifications show the serum levels of LDL-C (*n* = 6), HDL-C (*n* = 6), TG (*n* = 6), TC (*n* = 6), NEFA (*n* = 4) and fasting blood glucose (*n* = 10 for CTR group, *n* = 12 for HFD group, *n* = 14 for HFD+L and HFD+H group) in the mice of different groups. Error bars indicate SD; * or ^#^ *p* < 0.05, ** or ^##^ *p* < 0.01, *** or ^###^ *p* < 0.001 by one-way ANOVA. (**I**,**J**) Quantifications show serum activity of ALT and AST in the mice of different groups. Error bars indicate SD; * or ^#^ *p* < 0.05, by one-way ANOVA; *n* = 4.

While several factors contribute to the development and progress of NAFLD, obesity is reported to be the leading cause [28]. Thus, we first generated a high-fat-diet-induced obesity and NAFLD mouse model to study the roles of the SMRR polysaccharide in NAFLD. After 8 weeks of high-fat-diet feeding, mice gained more body weight than that of controls fed with a normal diet (Figure 2A). Then, the obese mice were divided into three groups: high-fat diet (HFD), high-fat diet and 10 mg/kg SMRR polysaccharide (HFD+L) and high-fat diet and 20 mg/kg SMRR polysaccharide (HFD+H) for another two weeks. We noticed that 20 mg/kg SMRR polysaccharide supplement (HFD+H) significantly reduced the body weight of obese mice (Figure 2A), and no difference in food uptake was observed among obese groups (Figure 2B). In addition, the levels of serum high-density lipoprotein (HDL-C) (Figure 2C), low-density lipoprotein (LDL-C) (Figure 2D), triglyceride (TG) (Figure 2E), cholesterol (TC) (Figure 2F), non-esterified fatty acid (NEFA) (Figure 2G) and fasting blood glucose (Figure 2H) were increased in obese mice and reduced after SMRR polysaccharide treatment, which all suggest an anti-obesity effect of the SMRR polysaccharide. To further evaluate the liver function, serum AST and ALT activity were quantified. We found that the high-fat diet increased the AST/ALT activity, suggesting liver dysfunction, while the SMRR polysaccharide significantly reduced ALT activity and slightly reduced AST activity, especially in the HFD+H group (Figure 2I,J). All these data reveal a protective effect of the SMRR polysaccharide against high-fat-diet-induced obesity and liver dysfunction.

### 2.3. SMRR Polysaccharide Attenuates High-Fat-Diet-Induced NAFLD Progress

**Figure 3:**
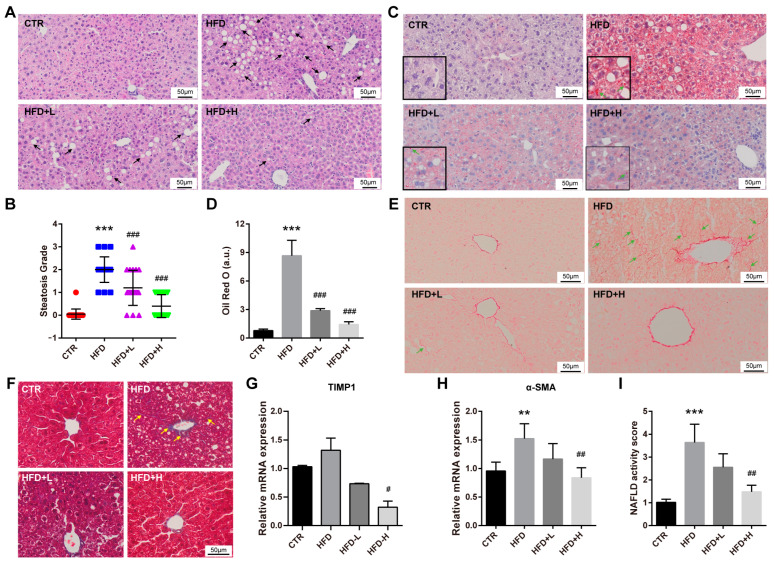
SMRR polysaccharide improves the progress of NAFLD. (**A**,**B**) Representative images of H&E staining and quantification show SMRR polysaccharide reverses the hepatocellular steatosis induced by high-fat diet. Error bars indicate SD; *** or ^###^ *p* < 0.001 by one-way ANOVA. Arrows indicate hepatocellular steatosis. Different colors in (**B**) represent different groups. (**C**,**D**) Representative images of Oil Red O staining and quantification show SMRR polysaccharide reverses the hepatic lipid accumulation induced by high-fat diet. Error bars indicate SEM; *** or ^###^ *p* < 0.001 by one-way ANOVA; a.u., arbitrary units. (**E**,**F**) Sirius Red staining (**E**) and Masson staining (**F**) show SMRR polysaccharide reverses the hepatic fibrosis induced by high-fat diet. Arrows indicate hepatic fibrosis. (**G**,**H**) qRT-PCR results show SMRR polysaccharide reverses the expressions of hepatic TIMP1 (*n* = 3) and α-SMA (*n* = 4) induced by high-fat diet. Error bars indicate SEM; ^#^ *p* < 0.05, ** or ^##^ *p* < 0.01 by one-way ANOVA. (**I**) Quantification shows SMRR polysaccharide reverses the NAFLD activity score induced by high-fat diet. Error bars indicate SEM; ^##^ *p* < 0.01, ** *p* < 0.001 by one-way ANOVA; *n* = 4; “*” stands for CTR vs. HFD, “#” stands for HFD vs. HFD+H.

To confirm the protective effect of the SMRR polysaccharide in liver dysfunction or injures at histopathological level, H&E staining was first performed. We found hepatocellular steatosis, a typical histological feature of NAFLD, in obese mice of the HFD group, while mice from SMRR polysaccharide supplement groups displayed much more normal hepatic histological morphology and steatosis grade, especially the mice from the HFD+H group (Figure 3A,B). Consistently, we noticed mice from SMRR polysaccharide supplement groups had fewer and smaller lipid droplets in the liver than those of mice from the HFD group (Figure 3C,D), shown by Oil Red O staining that stains lipid and triglyceride contents. Fibrosis is another hepatic histological feature of NAFLD [29]. Therefore, the collagen deposition in the liver was then detected by Sirius Red staining and Masson staining, finding clearly visible signs of those in the liver of mice from HFD group but not the mice from SMRR polysaccharide supplement groups, especially the HFD+H group (Figure 3E,F). In addition, the hepatic expressions of TIMP1 and α-SMA that related to hepatic fibrosis [30,31] were also increased in the mice from the HFD group but were suppressed in the mice from SMRR-polysaccharide-supplied groups (Figure 3G,H). Finally, combining the results from H&E staining, we calculated the NAFLD activity score (NAS) [32] of the livers and found that the SMRR polysaccharide significantly reduced the NAS of mice treated with the high-fat diet (Figure 3I), which suggested an improvement in the progress of NAFLD. Collectively, these results show that the SMRR polysaccharide could protect the liver from high-fat-diet-induced injures and attenuate the NAFLD progress.

### 2.4. SMRR Polysaccharide Ameliorates Defects in Gut Structure and Permeability Induced by High-Fat Diet

**Figure 4:**
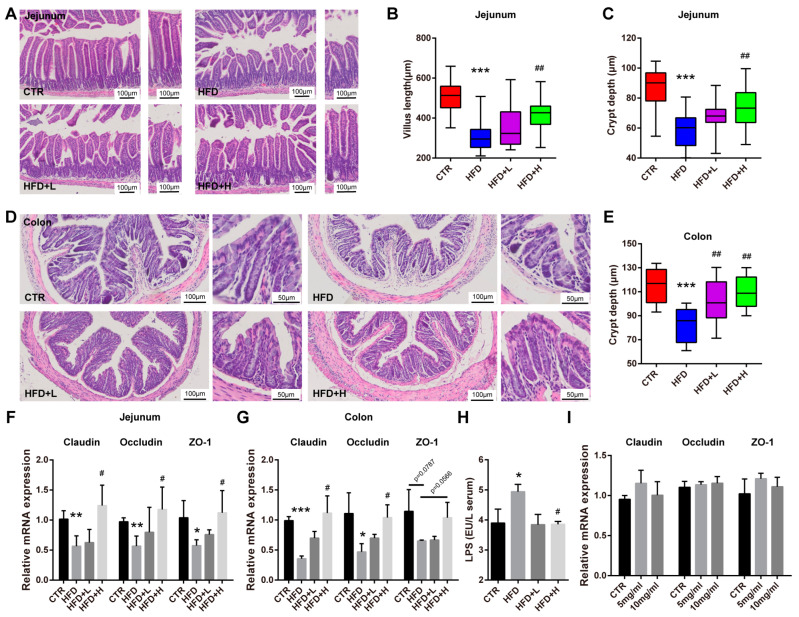
SMRR polysaccharide ameliorates defects in gut structure and permeability induced by high-fat diet. (**A**–**C**) Representative images of H&E staining and quantifications show SMRR polysaccharide reverses the defects in villus and crypt of jejunum induced by high-fat diet. Error bars indicate Mix to Max; *n* = 5; *** *p* < 0.001, ^##^ *p* < 0.01 by one-way ANOVA; “*” stands for CTR vs. HFD, “#” stands for HFD vs. HFD+H. Different colors in (**B**,**C**) represent different groups. (**D**,**E**) Representative images of H&E staining and quantification show SMRR polysaccharide reverses the defects in crypt of colon induced by high-fat diet. Error bars indicate Mix to Max; *n* = 5; *** *p* < 0.001, ^##^ *p* < 0.01 by one-way ANOVA. Different colors in (**E**) represent different groups. (**F**,**G**) qRT-PCR results show expressions of Claudin, Occludin and ZO-1 in jejunum and colon of mice from different groups. Error bars indicate SEM; * or ^#^ *p* < 0.05, ** or ^##^ *p* < 0.01, *** *p* < 0.001 by one-way ANOVA; *n* = 4. (**H**) Quantification shows serum LPS levels in the mice from different groups. Error bars indicate SD; *N* = 3; * or ^#^ *p* < 0.05 by one-way ANOVA; (**I**) qRT-PCR shows no effects of SMRR polysaccharide on the expressions of Claudin, Occludin and ZO-1 in IPEC-J2 cells. Error bars indicate SEM; *n* = 3.

Obviously, polysaccharides as macromolecules cannot directly function in the liver, and the intestine is reported to be their major target organ. In addition, gut microbiota, gut immune response and gut permeability are implicated in polysaccharides’ function [33]. Intestinal defects and injures are observed under high-fat diet treatment and obese condition [34,35]. Thus, we wondered if the protective effects of the SMRR polysaccharide on NAFLD are mediated by the gut–liver axis. With H&E staining, we noticed remarkable histopathological–structural defects of the jejunum from the small intestine and colon from the big intestine in mice fed with a high-fat diet, including decreased villus length and crypt depth (Figure 4A–E). Unsurprisingly, we found that the mice from SMRR polysaccharide supplement groups, especially the HFD+H group, displayed much more normal histopathological structures in the jejunum and colon (Figure 4A–E), suggesting better intestinal function. As the largest mucosal surface that provides an interface between the host and the external environment, the intestinal epithelium functions as a barrier to protect against toxic factors [36]. Tight junctions mediated by integral membrane proteins and junctional complex proteins are critical for intestinal epithelium barrier function, defects in which result in intestinal permeability dysfunction and are implicated in many human diseases, including obesity and NAFLD [37,38,39]. In the mice from the HFD group, we detected significantly decreased expressions of integral membrane proteins (Claudin and Occludin) and junctional complex protein ZO-1 in both the jejunum and colon (Figure 4F,G), which revealed defects in intestinal permeability. Consistent with this, elevated serum LPS concentration was detected in these mice (Figure 4H). After the consumption of the SMRR polysaccharide, the intestinal expressions of these genes increased and the serum LPS concentration decreased, especially in the mice of the HFD+H group (Figure 4F–H), which displayed a protective effect of the SMRR polysaccharide on intestinal permeability. However, we found that the SMRR polysaccharide could not promote the expressions of tight-junction-related genes (Claudin, Occludin and ZO-1) in cultured intestinal epithelium cells (Figure 4I), the suggesting SMRR polysaccharide displays its function in an indirect way.

### 2.5. SMRR Polysaccharide Attenuates Obesity-Induced Inflammation in Liver and Intestine

**Figure 5:**
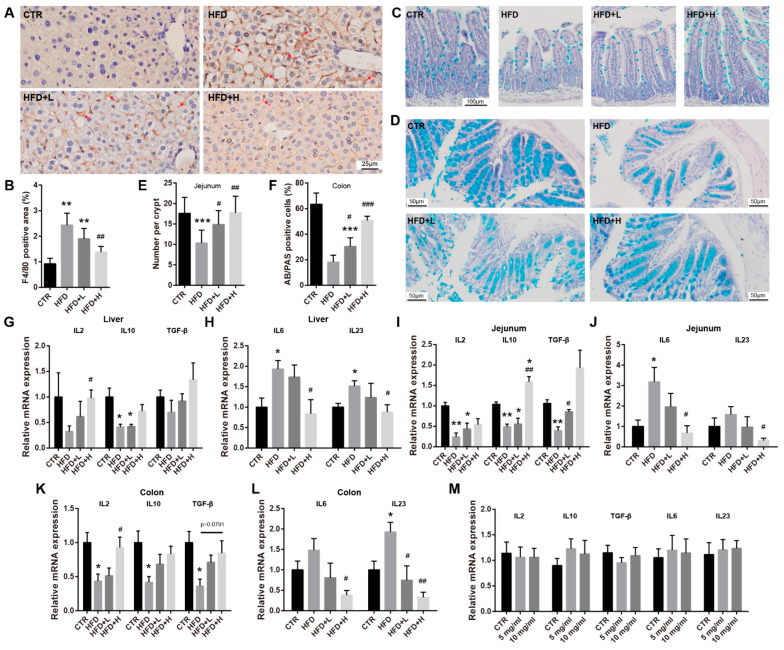
SMRR polysaccharide ameliorates inflammation in gut–liver axis induced by high-fat diet. (**A**,**B**) Representative images and quantification of F4/80 labeling in liver of each group of mice. Error bars indicate SEM; *n* = 4; ** or ^##^ *p* < 0.0 by one-way ANOVA. Arrows indicate F4/80 positive signals; “*” stands for CTR vs. HFD, “#” stands for HFD vs. HFD+H. (**C**–**F**) Representative images of AB-PAS staining and quantifications showing goblet cells in jejunum (**C**) and colon (**D**) of mice from different groups. Error bars indicate SD; *n* = 5; * or ^#^ *p* < 0.05, ^##^ *p* < 0.01, *** or ^###^ *p* < 0.001 by one-way ANOVA. (**G**–**L**) qRT-PCR results showing the expressions of anti-inflammatory genes (IL2, IL10 and TGF-β) and pro-inflammatory genes (IL-6 and IL23) in the liver, jejunum and colon of mice from different groups; *n* = 3; * or ^#^ *p* < 0.05, ** or ^##^ *p* < 0.01, *** or ^###^ *p* < 0.001 by one-way ANOVA. (**M**) qRT-PCR showing no effects of SMRR polysaccharide on the expressions of anti- or pro-inflammatory genes in JPEC-J2 cells; *n* = 4.

Inflammation is a common pathological feature induced by obesity that is implicated in the crosstalk between the liver and gut [19,40], while hepatic inflammation plays an important role in developing and progressing NAFLD [41]. Thus, we further evaluated the function of the SMRR polysaccharide on obesity-induced liver and intestinal inflammation. With F4/80 immunohistochemistry labeling that displays macrophage recruitment and activation, we found remarkably increased F4/80 signals in the liver of mice from the HFD group compared with the CTR group (Figure 5A,B). In the mice of the HFD+H group, we found much fewer F4/80 signals after SMRR polysaccharide supplementation, suggesting attenuated liver inflammation (Figure 5A,B). In addition to this, we also evaluated the inflammation in the intestine. With AB-PAS staining to quantify the goblet cells that are important for intestinal immunity, we found a significantly decreased number of goblet cells in the jejunum and colon of high-fat-diet-induced obese mice, while a re-balance of this defect was observed in the mice of SMRR polysaccharide supplement groups, which revealed attenuated intestinal immune response of obese mice with SMRR polysaccharide treatment (Figure 5C–F). Consistently, with qRT-PCR, we observed a decreased trend in the expressions of anti-inflammatory genes (IL2, IL10 and TGF-β) and increased trend in the expressions of pro-inflammatory genes (IL-6 and IL23) in the liver, jejunum and colon of mice fed with a high-fat diet, while SMRR polysaccharide supplementation reversed these trends (Figure 5G–L). These data collectively show benefits of the SMRR polysaccharide on improving inflammation of the gut–liver axis of obese mice. Interestingly, as with tight-junction-related genes, we also detected no effects of the SMRR polysaccharide on the expressions of inflammation-related genes in cultured cells (Figure 5M), which also indicates other pathways for the SMRR polysaccharide regulating the gut–liver axis and its related obesity and NAFLD progress.

### 2.6. SMRR Polysaccharide Regulates Gut–Liver Axis through Modulating Gut Microbiota

**Figure 6:**
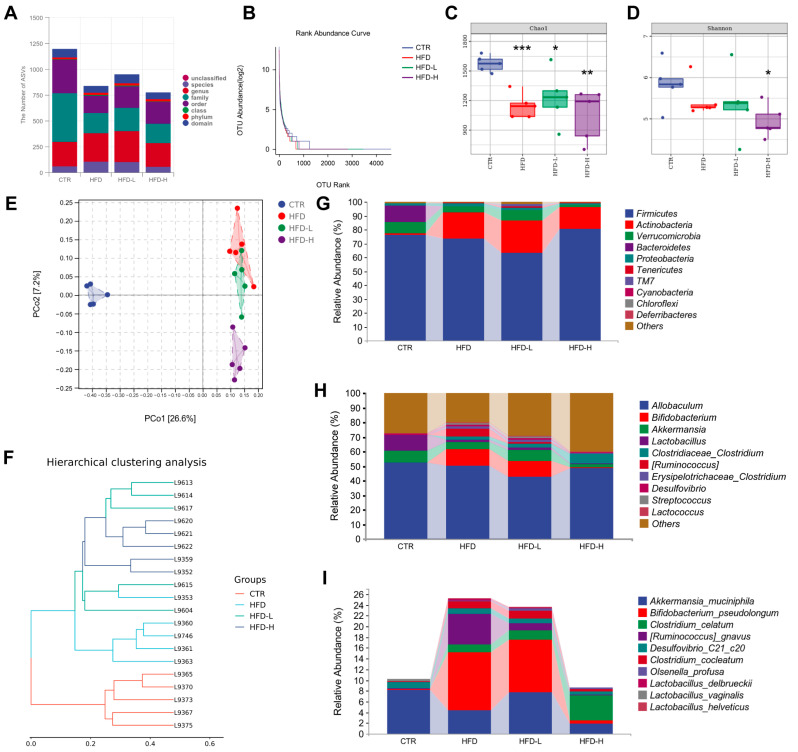
SMRR polysaccharide modulates the composition and structure of gut microbiota. (**A**) Quantification shows decreased numbers of ASVs of gut microbiota in HFD, HFD+L and HFD+H, compared with CTR. (**B**) OTU Rank–Abundance Curves of gut microbiota for different groups. (**C**) Bacterial community richness measured by Chao1 index in different groups; “*” stands for the comparation with CTR; * *p* < 0.05, ** *p* < 0.01, *** *p* < 0.001 by one-way ANOVA. (**D**) Bacterial community diversity measured by Shannon index in different group. * *p* < 0.05 by one-way ANOVA; (**E**) Unweighted UniFrac Principal Coordinate Analysis by bacterial microbiota. CTR shows clear separation with the other groups. (**F**) Bray Curtis cluster tree shows samples from CTR, HFD, and HFD+L trend to cluster together. (**G**–**I**) Microbial composition at the phylum, genus and species level. Groups are represented along the horizontal axis and relative abundance is denoted by the vertical axis.

More and more evidence demonstrates that the gut microbiota is implicated in regulating food energy extraction, lipid metabolism, intestinal immune response, etc., the processes of which are important for the development of obesity and its related comorbidities [42,43]. As the SMRR polysaccharide is not directly involved in regulating intestinal tight junction and immune response, we assessed if the gut microbiota was its target. By sequencing the gut bacterial 16S rRNA V3+V4 region, the intestinal microbiota composition was evaluated. As shown in Figure 6A, decreased total numbers of ASVs were observed in mice from both the HFD group and SMRR-polysaccharide-supplied groups. Then, we calculated the Rank–Abundance Curve [44], Chao1 [45] and Shannon [46] index to evaluate the richness and diversity of the intestinal microbiota, finding them reduced in mice from both the HFD group and SMRR-polysaccharide-supplied groups (Figure 6B–D). To determine the dissimilarity in the structure of gut microbiota among different groups, UniFrac distance-based Principal Coordinate Analysis (PCoA) [47] (Figure 6E) and Bray Curtis cluster analysis [48] (Figure 6F) were performed. We found that both HFD and polysaccharide-supplied groups displayed distinct clustering of microbiota composition compared to the CTR group, while there was an overlap of the clustering between the HFD and HFD+L group (Figure 6E). Consistently, in hierarchical clustering analysis, we found samples from the CTR, HFD and HFD+H group trended to cluster together and were away from each other, while samples from the HFD+L group were scattered clustered (Figure 6F).

Furthermore, we focused on the taxonomic distribution of the abundant bacteria from phylum, genus and species levels. At phylum level, *Firmicutes* (76.0%), *Bacteroidetes* (12.0%) and *Verrucomicrobia* (8.2%) represent most of the relative abundance of the gut microbiota in the CTR group, while *Firmicutes*, *Actinobacteria and Verrucomicrobia* represent most of that in HFD (73.5%, 18.8% and 4.4%) and polysaccharide-supplied groups (63.5%, 22.8% and 7.8% for HFD+L; 80.5%, 15.5% and 1.9% for HFD+H) (Figure 6G). The increased *Actinobacteria* relative abundance and decreased *Bacteroidetes* relative abundance, in HFD and SMRR-polysaccharide-supplied groups, may be a consequence of the consumption of a high-fat diet, according to previous studies [49,50]. At the genus level, *Allobaculum* was the most enriched genera in all groups, followed by Lactobacillus (10.3%) and *Akkermansia* (8.2%) in the CTR group, Bifidobacterium (11.4%) and *Ruminococcus* (5.7%) in the HFD group, Bifidobacterium (10.9%) and *Akkermansia* (7.8%) in the HFD+L group, and *Clostridiaceae_Clostridium* (6.3%) and *Akkermansia* (1.9%) in the HFD+H group (Figure 6H). At the species level, we observed higher relative abundance of *Bifidobacterium_pseudolongum*, *Ruminococcus_gnavus*, *Clostridium_celatum*, *Clostridium_cocleatum* and Desulfovibrio_C21_c20 in the HFD and HFD+L group, and *Clostridium_celatum* in HFD+H group, when compared to the CTR group (Figure 6I). All these data reveal an effect of the SMRR polysaccharide on modulating the composition and structure of gut microbiota.

**Figure 7:**
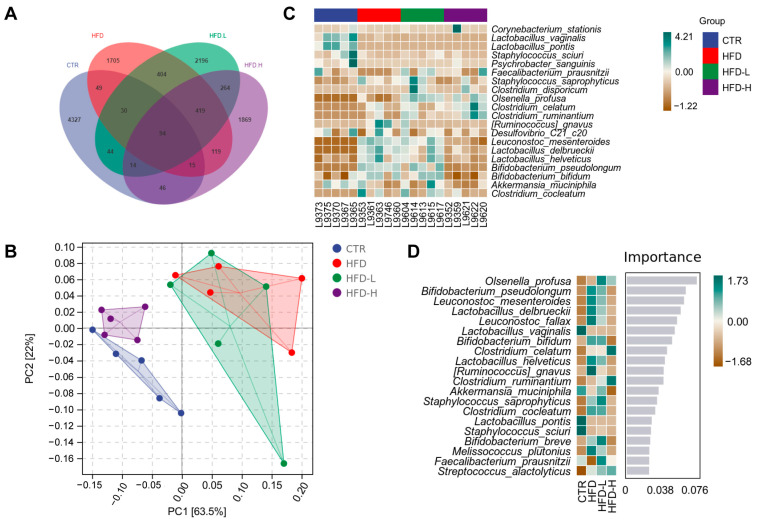
Comparative analysis of the gut microbiota in response to SMRR polysaccharide supplement. (**A**) Venn diagram showing the unique and shared ASVs from different groups. (**B**) The diagram of Principal Component Analysis (PCA) displaying close clustering between CTR and HFD+H group, while HFD is close to HFD+L. (**C**) Heatmap depicting the relative abundances of the 20 bacterial species significantly enriched in different samples of different groups. (**D**) Heatmap of Random Forest Classifier showing the relative abundances of the top 20 bacterial species of most importance in different groups.

To identify the key phylotypes of gut microbiota implicated in the SMRR polysaccharide’s function, we further compared the relative abundance of gut microbiota among different groups. With a Venn diagram, we found that there were only 95 ASVs shared by all groups (Figure 7A), and the Principal Component Analysis (PCA) analysis at the species level displayed close clustering between the CTR and HFD+H group (Figure 7B). To further analyze differences in species abundance, a heat map was generated with 20 bacterial species of the highest average abundance for species composition analysis. We found that the supplement with the SMRR polysaccharide (especially 20 mg/kg) generated changes in 15 bacterial species compared with the HFD group. Among these changes, nine were reversed to the same direction of those observed within the CTR group (Figure 7C), and some of these bacterial species were reported to be correlated with obesity and intestinal inflammation, such as *Ruminococcus_gnavus* [51,52], *Desulfovibrio_C21_c20* [53,54], *Bifidobacterium_pseudolongum* [51] and *Clostridium_cocleatum* [55,56]. Consistently, most of these changed bacterial species were also highlighted by a Random Forest Classifier [57,58], which identifies the marker species and calculates their importance (Figure 7D). Collectively, these data indicate that SMRR polysaccharide supplementation improves HFD-induced gut microbiota dysbiosis, which would be beneficial for attenuating NAFLD through the gut–liver axis.

## 3. Discussion

Current knowledge reveals that gut microbiota is implicated in the development of obesity and its related comorbidities [43], thus more and more studies focus on the characterization of biomolecules modulating the gut microbiota to treat these disorders. In this study, we isolated a polysaccharide from a TCM—*Salviae miltiorrhizae* Radix et Rhizoma (Danshen)—and reported the benefits of it on improving obesity and its related NAFLD. *Salviae miltiorrhizae* Radix et Rhizoma has been widely used in clinics of Asian countries, not only traditionally for the treatment of coronary heart disease (CHD), angina pectoris, myocardial infarction and atherosclerosis, hepatic injury, etc. [22,59], but also used in the treatments of tumors and neurological and metabolic disorders in modern medicine [60,61,62]. With the advancement of isolating and analyzing macromolecular compounds, polysaccharides have recently been reported as one of the important active components of *Salviae miltiorrhizae* Radix et Rhizoma. Multiple studies showed a protective effect of polysaccharides from *Salviae miltiorrhizae* Radix et Rhizoma on improving acute liver injury in mice induced by LPS, D-galactosamine or the bacillus Calmette-Guerin vaccine [41,42,43,44,45], while Zhang et al. found that the *Salviae miltiorrhizae* Radix et Rhizoma polysaccharide could ameliorate insulin resistance induced by tert-butyl hydroperoxide [63]. These works well displayed the potential of *Salviae miltiorrhizae* Radix et Rhizoma polysaccharides in the treatment of metabolic and hepatic diseases. Our work extends this knowledge and provides strong evidence showing bioactivity of the *Salviae miltiorrhizae* Radix et Rhizoma polysaccharide improving obesity and ameliorating NAFLD, including reduced hepatocellular steatosis, hepatic fibrosis and inflammation, which lays an important foundation for its further development and utilization.

The human intestine contains diverse kinds of polysaccharides, but only a select few of which are used as energy and carbon sources [33,64]. Most of the polysaccharides obtained from vegetables, fruits, etc., act as dietary fiber, without degradation or absorption, moving to the intestine and encountering gut microbiota. Therefore, increasing evidence demonstrates that the intestine is the target organ of polysaccharides and modulating the homeostasis of the gut microbiota is how they work. On this basis, multiple theories based on the correlation between the gut and other organs have been established, including the theories of the gut–liver axis, gut–brain axis, gut–lung axis, etc., which have been applied to the treatment of corresponding diseases [2,65,66,67]. Some previous studies have reported functional plant polysaccharides displaying effects regulating the gut–liver axis. A *Rosa rugosa* polysaccharide and an *Echinacea purpurea* polysaccharide can protect acute alcoholic liver disease by enhancing the liver function through the gut–liver axis [68,69], while an *Ophiopogon* polysaccharide promotes the production of intestinal SCFAs that enhances the hepatic AMPK pathway affecting liver lipid accumulation [70]. In our study, consistent with previous works [71,72,73], we found that a high-fat diet impairs the intestinal epithelium structure, tight junction and permeability, and induces intestinal inflammation, which are all remarkably attenuated after the consumption of the SMRR polysaccharide. These results reveal that intestine is also the target organ for the SMRR polysaccharide, and improvements in intestinal structure and function provide a basis for the treatment of NAFLD based on the gut–liver axis.

In addition to improvements in intestinal histopathology, we also noticed the function of the SMRR polysaccharide regulating the homeostasis of gut microbiota. The SMRR polysaccharide changed the richness, diversity and structure of the gut microbiota, compared with those in high-fat-diet-induced obesity/NAFLD mice. In addition, some of these changes were reversed back to the direction exhibited by normal mice. Detailed analysis highlighted some bacterial species implicated in the SMRR-polysaccharide-modulated gut–liver axis. *Ruminococcus gnavus* is a mucin-degrading gut bacterium that plays a role in gut immune development [74], and *Clostridium_cocleatum* can degrade mucin, impair the gut barrier and induce inflammation [55]. Previous studies found increased intestinal abundance of *Ruminococcus_gnavus* [51,75] and *Clostridium_cocleatum* [55,76,77] in obese subjects; our study confirmed this and displayed the effect of SMRR polysaccharide reversing this enrichment, which may contribute to better intestinal function. We also observed increased abundance of *Bifidobacterium_pseudolongum* in obese mice, and decreased abundance of the same after SMRR polysaccharide supplementation. Bernardo et al. noticed increased abundance of *Bifidobacterium_pseudolongum* in obese rats [51], Zhao et al. reported a negative correlation between obesity and *Bifidobacterium_pseudolongum* abundance, while Audrey et al. found that the Western diet did not affect its abundance. These diverse results suggest that the abundance of *Bifidobacterium_pseudolongum* may not be a suitable indicator for obesity-related gut microbiota homeostasis, and the same situation was reported on *Desulfovibrio_C21_c20* [53,78,79] and *Akkermansia_muciniphila* [76,78,80]. Interestingly, in our study, we observed remarkable increased abundance of some bacteria from well-known probiotic genera—*Bifidobacterium*, *Lactobacillus* and *Leuconostoc*—in obese mice which was reversed by SMRR polysaccharide consumption, such as *Bifidobacterium_pseudolongum*, *Bifidobacterium_breve, Bifidobacterium_bifidum*, *Lactobacillus_delbrueckii*, *Lactobacillus_helveticus* and *Leuconostoc_mesenteroides.* While administration of these bacteria was reported to have anti-obesity effects [81,82,83,84,85], our work provide evidence from another side showing this may be a double-edged sword. In addition, according to our Random Forest Classifier analysis (Figure 7D), increased abundances of *Leuconostoc_fallax*, *Melissococcus_plutonius* and *Staphylococcus_saprophyticus* were also positively correlated with obesity, for the first time, which was also reversed by SMRR polysaccharide supplementation. All these data reveal the fact that the SMRR polysaccharide is an effective modulator contributing to the homeostasis of gut microbiota and its related intestinal function. With ameliorated intestinal permeability, the SMRR polysaccharide decreases the serum LPS, which is the major cause of obesity-induced liver inflammation, thus improves the progress of NAFLD through the gut–liver axis.

## 4. Materials and Methods

### 4.1. Isolation, Purification and Characterization of SMRR Polysaccharide

The isolation of polysaccharides was carried out based on the previous methods [86]. Briefly, the roots and rhizomes of *S. miltiorrhiza* were pre-extracted by 80% ethanol (*v*/*v*), and the dried residue was extracted with boiling water under the following conditions: 100 °C, solid–liquid ratio 1:40, 2 h, 3 times. Then, the water extracts were concentrated and dialyzed (cut off 3500 Da), followed by precipitation with 4-fold ethanol at 4 °C overnight. The precipitate was collected and lyophilized by using a freeze dryer (LGJ-10G, Beijing Sihuan Qihang technology Co., Ltd., Beijing, China), and named as crude SMRR polysaccharide.

Crude SMRR polysaccharide (300 mg) was dissolved in distilled water (20 mL), filtrated (0.45 μm) and applied into an anion-exchange column packed with DEAE- Sepharose Fast Flow (4.6 × 60 cm, Beijing Rui Da Heng Hui Science Technology Development Co. Ltd., Beijing, China). The neutral and acidic fractions were eluted with 1500 mL distilled water followed by 0–1.5 mol/L NaCl solution at a flow rate of 2 mL/min, respectively. The elution profiles of acidic fractions were monitored by the phenol–sulfuric acid method [87]. An acidic fraction was obtained after dialysis (cut off 3500 Da) and freeze-drying, named as SMRR-1, and was used for further studies.

SMRR-1 (20 mg) was dissolved in 5 mL distilled water and applied to a gel filtration through Sepharose 6FF matrix (2.5 cm× 100 cm, Beijing Rui Da Heng Hui Science Technology Development Co. Ltd.) at 0.5 mL/min. The eluent was collected in 5 mL/tube, and the presence of carbohydrate was monitored by the phenol–sulfuric acid test. One homogenous fraction, named SMRR polysaccharide, was collected and freeze-dried for further study.

The molecular weight of SMRR polysaccharide was determined under following chromatographic conditions: Waters Ultrahyrdrogel Linear gel column (300 × 7.8 mm) with Waters 2410 differential refractive detector and Waters 515 liquid chromatography pump were used; the mobile phase was 0.2 mol/L NaNO3 solution, pH = 6.0; flow rate was 0.6 mL/min; column temperature was 40 °C; the injection volume was 20 μL. The dex-tran standards (molecular weight range was 2500~5,348,000 Da, Sigma-Aldrich, St. Louis, MO, USA, Item NO. 34036258) and SMRR polysaccharide were dissolved in the mobile phase. Sample solutions were injected after the system was stable, and the resulting chromatogram was recorded. The weight-average molecular weight (*M*_w_), number-average molecular weight (Mn) and their distribution equivalents were obtained by the standard curve.

An amount of 1 mg SMRR polysaccharide was hydrolyzed by anhydrous 3 M HCl methanol at 80 °C for 20 h, and 100 μg mannitol was used as internal standard. The volatile trimethylsilyl derivatives (TMSi) of monosaccharides were obtained by derivatization with hexamethyldisilazane (HDMS) and trimethylchlorosilane (TMCS) [88], and were further analyzed using capillary gas chromatography (Carlo Erba 6000, Vega Series 2, J & W scientific Inc, Carlo Erba, Italy) with ICU 600 program. Identification of the monosaccharides was based on their retention times and the relative amount of each monosaccharide was calculated based on peak integration compared with monosaccharide standards [89,90].

### 4.2. Animals and Treatments

All mouse work was conducted in accordance with the Animal Care and Use Committee guidelines of Sichuan Agricultural University. All mice were housed under SPF conditions in standard individually ventilated cages at 20–22 °C, with a 12 h light/12 h dark cycle, 50–70% humidity and ad libitum access to standard chow and water. To generate the obesity/NAFLD mouse model, 50 4-week-old C57/BL6 male mice were ordered from Vital River Laboratory Animal Technology Co. Ltd., Beijing, China. After one week acclimatization, we divided these mice into two groups. First, one group was supplied with a normal diet (*n* = 10, ND, MD17131, Medicience, Yangzhou, Jiangsu, China). Second, the other was supplied with a high-fat diet (*n* = 40, HFD, 45%fat, MD12032, Medicience, Yangzhou, China). After 8 weeks of feeding, we divided the HFD group into three groups: HFD (*n* = 12, supplied with high-fat diet and solvent), HFD+L (*n* = 14, supplied with high-fat diet and 10 mg kg^−1^ SMRR polysaccharide), HFD+H (*n* = 14, supplied with high-fat diet and 20 mg kg^−1^ SMRR polysaccharide). The solvent (saline) or polysaccharide solution was supplied daily for 14 days intragastrically, and the body weight was measured weekly. At the end of the experiment, half of the mice from all groups were fasted overnight and the blood was collected through cardiac blood collection for the assays of serum glucose and LPS, blood biochemistry and liver function tests; then, these mice were euthanized, and liver and intestinal samples were isolated for biochemical assays and qRT-PCR, while the fresh feces collected from the cecum were stored at −80 °C and sent to Personalbio (Shanghai, China) for gut microbiota analysis. The left half of the mice were transcardially perfused with PBS first, and the samples were collected for staining.

### 4.3. Histological Staining

Samples were fixed in 4% paraformaldehyde solution and embedded in paraffin. Then, 5 μm sections were made and mounted on slides for staining with hematoxylin and eosin (H&E, G1120, Solarbio, Beijing, China), Masson (G1340, Solarbio, Beijing, China), Sirius Red (G3632, Solarbio, Beijing, China) and AB-PAS (G1285, Solarbio, Beijing, China), according to the manufactures’ instructions. For Oil Red O staining, frozen sections of the livers were first obtained, and the staining was performed according to the manufacturer’s instructions (G1260, Solarbio, Beijing, China), while the quantification was performed according to a previous protocol [91].

With H&E staining, hepatocellular steatosis was graded from 0 to 3 based on the percentage of hepatocytes involved (0 = <5%; 1 = 5–33%; 2 = 33–66%; 3 = >66%), according to a previous study [92]. The NAS score was calculated by steatosis (0–3), lobular inflammation (0−3) and ballooning (0−2), also according to a previous study [32].

### 4.4. Immunohistochemical Staining

A SADB-POD kit (Boster, SA2002, Wuhan, China) was used to perform the immunohistochemical staining, according to the manufacturer’s instructions. First, deparaffinization and rehydration of paraffin embedded sections were performed. Second, the endogenous peroxidase activity was blocked by 3% H_2_O_2_ (20 min, RT). Third, the high-pressure-mediated antigen retrieval processes were performed using Citrate Buffer (pH = 6.0) after washing them with PBS. Fourth, tissue sections were blocked with the blocking buffer (1 h, RT, 10% goat serum in PBS + 0.1% Triton X-100, if a permeabilization was needed). Fifth, the tissue sections were incubated with primary antibodies (in PBS with 1% goat serum, 4 °C, overnight). Then, they were incubated with biotin labeling secondary antibody and SABC, in turn (RT, 30 min), after being washed three times with PBS. Finally, color development was performed with DAB, and the slides were counterstained with hematoxylin. Antibodies used in this study are listed below: F4/80 (Rabbit pAb,DF2789, Affinity, Jiangsu, China, 1:200).

### 4.5. Quantitative Realtime PCR

Total RNA from liver and intestine were extracted using Animal Total RNA Isolation Kit (RE-03014, Foregene, Chengdu, China). Then, ~1 µg RNA was used for the reverse transcription using RT EasyTM II (With gDNase) (RT-01032, Foregene, Chengdu, China) with the following conditions: 42 °C for 25 min and 85 °C for 5 min. Finally, RT-qPCR was performed with final concentrations of 2 ×TB Green Premix DimerEraser (Takara Biomedical Technology (Beijing) Co., Ltd., Beijing, China), and 0.3 mM forward and reverse primers in 25 μL, using the following conditions: 95 °C for 30 s; 40 cycles of 95 °C (5 s) and 60 °C (30 s) in the Bio-Rad CFX96 Real-Time Detection System (Bio-Rad, Hercules, CA, USA). The relative gene expression was normalized to internal control as β-Actin. Primers used in this study are listed below (Table 1).

### 4.6. Cell Culture

Intestinal porcine epithelial cells (IPEC-J2) were cultured under an atmosphere of 5% CO2 at 37 °C in Dulbecco’s modified Eagle’s medium (DMEM, Gibco, Waltham, MA, USA) with 10% fetal bovine serum (FBS; Gibco) and 1% penicillin–streptomycin (Gibco). To test the effects of SMRR polysaccharide on gene expressions, the cells were treated with 5 mg/mL and 10 mg/mL SMRR polysaccharide for 24 h. Then, the cells were harvested and subjected to Quantitative Realtime PCR analysis.

### 4.7. Enzyme-Linked Immunosorbent Assay (ELISA)

The concentrations of LPS were determined for serum using ELISA kits according to the manufacturer’s instructions (Ml85252-J, MlBio, Shanghai, China). Briefly, ELLISA plate was first prepared at RT for 1 h before use. Second, 50 μL serum samples or standard substance was added into the wells of plate followed by 100 μL HRP-labeled detecting antibody and incubated at 37 °C for 30 min. Then, 50 μL substrate A and B were added after five washes with a wash buffer. Finally, 50 μL stop buffer was added and the OD values were quantified at 450 nm with a microplate reader (Thermo Fisher Scientific, Waltham, MA, USA).

### 4.8. Gut Microbiota Analysis

Total genomic DNA samples were extracted from fecal samples stored at −80 °C using the OMEGA Soil DNA Kit (M5635-02) (Omega Bio-Tek, Norcross, GA, USA), according to the manufacturer’s instructions, and stored at −20 °C. A NanoDrop NC2000 spectrophotometer (Thermo Fisher Scientific, Waltham, MA, USA) and agarose gel electrophoresis were used to measure the quantity and quality of the extracted DNAs, respectively.

The V3/V4 regions of the 16S rRNA genes were amplified by PCR using the following primers: forward primer 338F (5’-ACTCCTACGGGAGGCAGCA-3’), reverse primer 806R (5’-GGACTACHVGGGTWTCTAAT-3’). Sample-specific 7 bp barcodes were incorporated into the primers for multiplex sequencing. Thermal cycling consisted of the following condition: 98 °C for 5 min (1 cycle), followed by 25 cycles of 98 °C for 30 s, 53 °C for 30 s and 72 °C for 45 s. The Vazyme VAHTSTM DNA Clean Beads (Vazyme, Nanjing, China) and the Quant-iT PicoGreen dsDNA Assay Kit (Invitrogen, Carlsbad, CA, USA) were used to purify and quantify the PCR products, respectively. Then, a high-throughput sequencing was performed using the Illlumina NovaSeq platform with NovaSeq 6000 SP Reagent Kit (500 cycles) at Shanghai Personal Biotechnology Co., Ltd. (Shanghai, China).

Microbiome bioinformatics were performed with QIIME2 2019.4 [93] with slight modification according to the official tutorials (https://docs.qiime2.org/2019.4/tutorials/) (Accessed on 11 March 2022). The raw sequence was demultiplexed using the demux plugin and the primers were cut with the cutadapt plugin [94]. The quality, filtering, denoising, merging and chimera removal of the sequences were performed according the DADA2 plugin, and the non-singleton amplicon sequence variants (ASVs) were aligned with mafft [95] to construct a phylogeny with fasttree2 [96]. Taxonomy was assigned to ASVs using the classify-sklearn naïve Bayes taxonomy classifier in feature-classifier plugin [97] against the SILVA Release 132 Database [98].

Sequence data analyses were mainly performed using QIIME2 and R packages (v3.2.0). ASV-level alpha diversity, including Chao1 richness estimator and Shannon diversity index, were calculated using the ASV table in QIIME2. ASV-level ranked abundance curves were generated to compare the richness and evenness of ASVs among samples. Beta diversity analysis was performed to investigate the structural variation of microbial communities across samples using UniFrac distance-based Principal Coordinate Analysis (PCoA) [47] and Bray Curtis cluster analysis [48]. The species-level compositional profiles were used to perform Principal Component Analysis (PCA) [99]. The shared and unique ASVs among groups were visualized by Venn diagram using R package “VennDiagram”, based on the occurrence of ASVs across groups regardless of their relative abundance [100]. Taxa abundances at the species level were statistically compared among samples or groups, visualized as heatmap, with the top 20 most rich bacterial species. Random Forest Analysis was applied to discriminate the samples from different groups using QIIME2 with default settings [57,58].

### 4.9. Statistical Analysis

Data represent the mean ± standard deviation (SD) or mean ± standard error of the mean (SEM). One-way ANOVAs were performed for the statistical significance analysis using GraphPad Prism software (Version 6.0, San Diego, CA, USA): * *p* < 0.05, ** *p* < 0.01, *** *p* < 0.01.

## 5. Conclusions

In summary, this study validates a prominent role of a polysaccharide from *Salviae miltiorrhizae* Radix et Rhizoma in regulating the homeostasis of gut microbiota, thereby providing beneficial effects against obesity, as it induced defects of intestinal permeability and inflammation, which ameliorated the hepatocellular steatosis and hepatic fibrosis to improve NAFLD through the gut–liver axis in high-fat-diet-fed mice.

## Figures and Tables

**Table 1:** Primers used in this study.

Gene	Primer	Sequence
mouse *β-actin* NM_007393	F	5′-AGAGGGAAATCGTGCGTGAC-3′
	R	5′-CAATAGTGATGACCTGGCCGT-3′
mouse *IL-2* NM_008366	F	5′-CCTGAGCAGGATGGAGAATTACA-3′
	R	5′-TCCAGAACATGCCGCAGAG-3′
mouse *IL-6* NM_031168	F	5′-CTTCCATCCAGTTGCCTTCTTG-3′
	R	5′-AATTAAGCCTCCGACTTGTGAAG-3′
mouse *IL-10* NM_010548	F	5′-AAGGGTTACTTGGGTTGCCA-3′
	R	5′-CCTGGGGCATCACTTCTACC-3′
mouse *IL-21* NM_001291041	F	5′-GCATGGAGAGGACCCTTGTC-3′
	R	5′-CTAATCAGGAGGCGATCTGGC-3′
mouse *IL-23* NM_031252	F	5′-ATGCTGGATTGCAGAGCAGTA-3′
	R	5′-ACGGGGCACATTATTTTTAGTCT-3′
mouse *TGF-β* NM_011577	F	5′-GTGTGGAGCAACATGTGGAACTCTA-3′
	R	5′-CGCTGAATCGAAAGCCCTGTA-3′
mouse *Claudin-1* NM_016674	F	5′-TGGTAATTGGCATCCTGCTG-3′
	R	5′-CAGCCATCCACATCTTCTGC-3′
mouse *Occludin* NM_008756	F	5′-GTACCCACCAGTGACCAACA-3′
	R	5′-GTTGCTGGAGCTTAGCCTGT-3′
mouse *ZO-1* NM_001163574	F	5′-CGAGGCATCATCCCAAATAAGAAC-3′
	R	5′-TCCAGAAGTCTGCCCGATCAC-3′

## Data Availability

Source data are provided in this paper and are available from the corresponding author upon reasonable request.
